# “Doctored: fraud, arrogance, and tragedy in the quest to cure Alzheimer’s”: an important new book with direct relevance to *GeroScience*

**DOI:** 10.1007/s11357-025-01661-2

**Published:** 2025-04-22

**Authors:** Csaba Szabo

**Affiliations:** Section of Pharmacology, Department of Oncology, Microbiology and Immunology, Faculty of Science and Medicine, University of Fribourg, Fribourg, Switzerland

**Keywords:** Alzheimer's disease, Amyloid, Retractions, Academia, Brain, Neurodegeneration

Charles Piller, a seasoned investigative journalist, has spent years exposing fraud and misconduct in neuroscience research, particularly Alzheimer’s disease, with several of his explosive articles published in *Science* [1–11]. Piller uncovered serious issues related to various beta-amyloid antibody clinical trials, revealed manipulated data from Cassava Biosciences and its affiliated CUNY investigator Hoau-Yan Wang, and dismantled the legitimacy of Sylvain Lesné and Karen Ashe’s now-retracted *Nature* paper on a special form of beta-amyloid. Other exposed figures include Berislav Zlokovic (ZZ Therapeutics) and Eliezer Masliah (NIH), whose work contained manipulated data that “supported” various patent filings and clinical trials. Piller’s work has sent shockwaves through the scientific community, highlighting the devastating consequences for patients and the waste of public funds. It triggered investigations into misconduct, leading to retractions and firings.

His new book, *Doctored: Fraud, Arrogance, and Tragedy in the Quest to Cure Alzheimer’s* [12], expands upon his investigative reports, revealing systemic issues that have plagued the field for decades. The book places these isolated reports within the broader context of the global Alzheimer’s research ecosystem. It documents the Cassava Biosciences debacle, Biogen’s marginally effective amyloid antibody programs, the fraudulent *Nature* paper, and highlights the broader issues of perverse incentives in academia, publishing, and biotech. Piller exposes what he unapologetically calls the “amyloid mafia,” a powerful cabal that controls Alzheimer’s research by prioritizing novelty over replication and marginalizing dissenters. He also exposes the publishing industry’s reluctance to retract fraudulent work, biotech’s greed, and the FDA’s many questionable decisions. Piller spares no one. He documents how regulatory agencies, entangled with industry interests, consistently fail to protect public health. The infamous “revolving door” between pharma executives and regulatory officials allows companies to evade accountability while cashing in on marginal drugs. Despite expert recommendations, the FDA has repeatedly approved dubious Alzheimer’s treatments while dismissing legitimate concerns. For instance, Cassava Biosciences’ clinical trials were allowed to proceed despite overwhelming evidence of misconduct and a Citizen’s Petition calling for their suspension.

A particularly striking section of the book, *More Blots*, details how Piller, Vanderbilt professor Matthew Schrag, and a network of scientific sleuths analyzed numerous Alzheimer’s research papers. Their findings reveal a shocking number of manipulated images, implicating dozens of high-profile researchers. For example, Masliah’s problematic papers underpin more than 200 patents used by multiple companies, some of which have ongoing clinical programs. The ramifications are staggering: billions in research funding wasted, careers built on false premises, and patients subjected to ineffective or harmful treatments.

Piller highlights those working to expose these issues, including Schrag, and the “science sleuths” Elisabeth Bik, David Bimler, Sholto David, Mu Yang, and Leonid Schneider. These dedicated individuals have played a crucial role in identifying fraudulent work, often at considerable personal and professional risk. Their efforts underscore the reality that scientific self-correction is not an automatic process but one that requires constant vigilance.

For most readers, *Doctored* will obliterate any remaining faith in the amyloid hypothesis. Piller suggests that the entire field may be built on shaky foundations, with fraudulent papers reinforcing preexisting dogmas rather than legitimate science. He cites Nobel laureate Thomas Südhof, who argues that many scientists producing fraudulent research are merely conforming to overblown dominant paradigms, many of which are based on shaky original data. By the book’s final chapter, Piller writes “…even if amyloid plays a role*…*”—a tacit acknowledgment that the theory’s credibility has been severely undermined. In turn, he explores alternative theories of neurodegeneration, giving voice to researchers who have long been marginalized for challenging the amyloid orthodoxy.

*Doctored* is essential reading for every biomedical scientist. It is particularly important for those in the broader scientific community who still regard fraud and bias as isolated incidents rather than systemic problems. It should also be required reading for policymakers, funders, and journal editors—those who hold the power to implement reforms but often remain oblivious to the scale of misconduct. Perhaps most importantly, it should be read by patients and their families, who deserve to know the truth about the treatments they are offered and the corporate and scientific forces shaping them. If this book accomplishes anything, it should be to shatter the illusion that Alzheimer’s research is on solid footing and to spark a long-overdue reckoning in the field. The evidence is overwhelming: systemic fraud, entrenched conflicts of interest, and regulatory failure have infiltrated Alzheimer’s research for decades.

It can no longer be claimed that misconduct and fraud in biomedical research are rare events or that those who engage in these activities are merely a few “bad apples.” Whole books have been published exposing the deep-seated problems in the global biomedical research ecosystem, highlighting the problem’s magnitude, the enormous sums wasted, and the perverse incentives and systemic failings that fuel the system [13–17]. *Doctored* merely highlights one particular aspect of a far wider problem. Tragically, even this “particular aspect” has enormous socioeconomic implications, when we consider that Alzheimer’s disease and other dementias are estimated to cost the world economy 14,513 billion dollars over the next 30 years [17]––unless new, effective therapies are introduced. It is therefore enormously important to enhance the reproducibility and translatability of Alzheimer’s research.

Well-intended manifestos, initiatives, strategies, rules, recommendations, criteria, guidelines, checklists, and beginners’ guides that all try to raise attention to what is usually rather euphemistically described as “reproducibility crisis” or “questionable research practices” or “scientific misconduct issues” regularly appear in various scientific journals [18–28]. Whether or not the standard recommendations––focusing, for example, on improved training and open data access––are working, however, is highly questionable. In any case, the question is no longer whether or not we are *aware of the problem*. Instead, the question now is whether the scientific community will finally take action.
